# Neck Active Movements Assessment in Women with Episodic and Chronic Migraine

**DOI:** 10.3390/jcm10173805

**Published:** 2021-08-25

**Authors:** Carina F. Pinheiro, Anamaria S. Oliveira, Tenysson Will-Lemos, Lidiane L. Florencio, César Fernández-de-las-Peñas, Fabiola Dach, Débora Bevilaqua-Grossi

**Affiliations:** 1 Department of Health Sciences, Ribeirão Preto Medical School, University of Sao Paulo, Ribeirão Preto 14049-900, Brazil; carinafp@usp.br (C.F.P.); siriani@fmrp.usp.br (A.S.O.); tenysson@fmrp.usp.br (T.W.-L.); 2 Department of Physical Therapy, Occupational Therapy, Rehabilitation and Physical Medicine, King Juan Carlos University, Alcorcón, 28922 Madrid, Spain; lidiane.florencio@urjc.es (L.L.F.); cesar.fernandez@urjc.es (C.F.-d.-l.-P.); 3 Department of Neurosciences and Behavioral Sciences, Ribeirão Preto Medical School, University of São Paulo, Ribeirão Preto 14049-900, Brazil; fabioladach@usp.br

**Keywords:** headache, cervical spine, motion, chronic pain, musculoskeletal pain

## Abstract

We aimed to compare movement parameters and muscle activity during active cervical spine movements between women with episodic or chronic migraine and asymptomatic control. We also assessed the correlations between cervical movement measures with neck-related disability and kinesiophobia. Women with episodic (*n* = 27; EM) or chronic (*n* = 27; CM) migraine and headache-free controls (*n* = 27; CG) performed active cervical movements. Cervical range of motion, angular velocity, and percentage of muscular activation were calculated in a blinded fashion. Compared to CG, the EM and CM groups presented a reduced total range of motion (*p* < 0.05). Reduced mean angular velocity of cervical movement was also observed in both EM and CM compared to CG (*p* < 0.05). Total cervical range of motion and mean angular velocity showed weak correlations with disability (r = −0.25 and −0.30, respectively; *p* < 0.05) and weak-to-moderate correlations with kinesiophobia (r = −0.30 and −0.40, respectively; *p* < 0.05). No significant correlation was observed between headache features and total cervical range of motion or mean angular velocity (*p* > 0.05). No differences in the percentage of activation of both flexors and extensors cervical muscles during active neck movements were seen (*p* > 0.05). In conclusion, episodic and chronic migraines were associated with less mobility and less velocity of neck movements, without differences within muscle activity. Neck disability and kinesiophobia are negative and weakly associated with cervical movement.

## 1. Introduction

Migraine is a primary headache ranked as the second world cause of disability when considered years living with disability [1]. Migraine diagnostic criteria are defined by recurrent attacks lasting 4–72 h with headaches that are typically unilateral, pulsating, moderate or severe intensity, aggravated by routine physical activity, and also associated with nausea and/or photophobia and phonophobia [2].

Migraine is recognized as a complex clinical condition considering its variety of symptoms and its range of comorbidities [3,4,5]. The association of migraine with neck pain or with pain on manual examination of the upper cervical joints is one of these interactions that might contribute to its clinical complexity, negatively influencing the impact, treatment, and prognosis of migraine [4,6,7,8].

Reduced cervical range of motion (ROM) has been confirmed in patients with migraines by the most updated meta-analyses [9,10]. The relationship of this reduced ROM with the frequency of migraine attacks is still under debate. Some reports suggest that cervical ROM is only impaired in patients with chronic migraines [11,12], while others do not support this [13,14,15]. Further, cervical ROM may also be affected by kinesiophobia reported by patients with migraines [16,17], especially considering that individuals with migraines frequently experience pain aggravation due to head movements during migraine attacks [17]. Although it has not been previously analyzed in migraineurs, angular velocity during cervical ROM is also a parameter for movement analysis that could contribute to detecting sensorimotor alterations of the neck [18]. Previous studies have revealed a reduced velocity of cervical movements in individuals with neck pain compared with asymptomatic subjects [18,19,20].

Individuals with migraines also exhibit altered superficial muscle activity during isometric contractions. Benatto et al. observed reduced extensor/flexor muscle activity ratio during maximal voluntary isometric contractions in flexion in migraineurs compared to healthy controls [21]. Furthermore, patients with chronic migraine also present higher coactivation of neck extensors during isometric contraction in cervical flexion [22] and a craniocervical flexion test [23] than healthy controls. However, to date, no study has assessed cervical muscle activity during active neck movements in patients with migraines.

The current study aimed to assess kinematic data (cervical range of motion and angular velocity) and muscle activity during active cervical ROM (flexion, extension, lateral flexions, and rotations) comparing asymptomatic women with episodic or chronic migraine sufferers. We also aimed to determine the correlation of the kinematic data with neck-related disability and kinesiophobia. We hypothesized that women with migraines would exhibit different kinematic patterns and muscle activity than headache-free controls. A secondary hypothesis was that kinematic patterns would be associated with related- disability and kinesiophobia in migraine women.

## 2. Materials and Methods

### 2.1. Participants Selection

Women aged between 18 and 55 were recruited from the local population between January 2018 and August 2019 through advertisements via social media (Instagram^®^, Facebook^®^) and local university radio. Potential participants were diagnosed by a neurologist of a headache clinic for both migraine groups according to the third edition of the International Headache Society criteria [2]. The episodic migraine group consisted of women presenting 2 to 12 days of migraine attacks for at least three months [2]. Participants who presented ≥15 days of headache attacks/month, which, on at least eight days/month, had the features of migraine headache for more than three months, composed the chronic migraine group [2]. Women without a history of frequent headaches composed the control headache-free group. The previous history of neck pain was permitted in the control group.

Participants were excluded if they underwent anesthetic nerve block or received physical therapy the previous year, history of degenerative cervical conditions, history of trauma at the neck and face, or pregnancy. For participants within both migraine groups, we also excluded those presenting with a second concomitant headache diagnosis (i.e., cervicogenic headache or tension-type headache) or those treated with botulinum toxin or anesthetic blocks. The local ethics committee approved this study protocol (protocol number 12145/2016), and all participants signed the written informed consent before their inclusion.

### 2.2. Instrumentation

**Figure 1:**
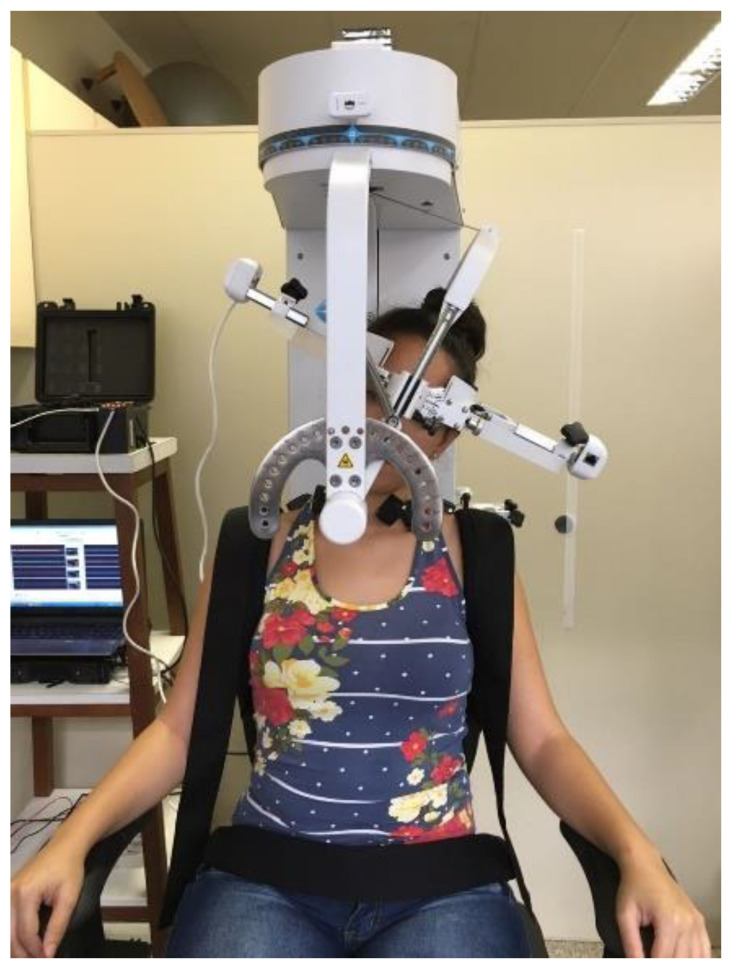
Representation of a participant’s position and stabilization on the Multi-Cervical Rehabilitation Unit (MCU).

The Multi-Cervical Rehabilitation Unit (MCU) (BTE Technologies, Inc.™, Hanover, USA) was used to assess active ROM. It is a fixed-frame device with a head assembly system (movable inner and outer head brace). It also contains an adjustable parameter at the seat to stabilize the individual and avoid compensations (Figure 1). The potentiometer was calibrated daily by first setting the outer brace (transverse plane) at 0°, 90° rotation to the left, and 90° to the right, and then by setting the inner brace (sagittal plane) at 0°, 100° flexion, and 100° extension. Measurement of the active ROM using this device has shown excellent reliability in people with neck pain and healthy participants (intraclass correlation coefficient ranging from 0.81 to 0.96) [24].

Surface EMG was acquired using the Trigno^TM^ Wireless System (CMRR of 80 dB, input impedance exceeding 1000 Ω, Delsys Inc. Boston, MA, USA). Each Trigno sensor comprises two parallel groups with two bars, each one (Ag-AgCl), with a fixed inter-electrode distance of 10 mm. Myoelectric signals were acquired, digitalized, amplified (gain = 300), band-pass filtered (20–450 Hz with 40 and 80 dB/dec), and sampled at 4 kHz per channel with a 16-bit resolution A/D by software EMGworks Acquisition (Delsys Inc. Boston, MA, USA).

An A/D converter board (USB-1616HS-BNC; Measurement Computer Corporation, Norton, MA, USA) was used to synchronize MCU and EMG data. The A/D converter board digitalized the electrical signals from the potentiometer previously amplified (MKTC5-10; MK Controle e Instrumentação, São Paulo, Brazil). It also received inputs from the Transistor-Transistor Logic (TTL) Trigger Module (Delsys Trigno; Delsys Inc. Boston, MA, USA). The digitalized data from MCU and Trigger Module were relayed to a customized MATLAB script and sampled at 2 kHz. The TrignoTM Wireless System and A/D converter board were connected to an external power supply (12 V, 9 Ah, rechargeable, GetPower) to avoid power grid noise.

### 2.3. Procedures

Clinical features of migraine, such as frequency of migraine episodes (days per month), the intensity of migraine attacks (numerical pain rate scale (NPRS), 0–10), and years with migraine were collected. Participants were also questioned about the self-rated presence of neck pain and its characteristics, including frequency, intensity, and time of onset. Finally, participants fulfilled the questionnaires Neck Disability Index (NDI) [25] and Tampa Scale for Kinesiophobia (TSK) [26]. The NDI is a 10-item questionnaire widely used to assess neck pain-related disability. Individual items are scored, and the total score can range from 0% to 100% [27]. The NDI has excellent reliability (intraclass correlation coefficient 0.86) [28] and internal consistency (Cronbach alpha 0.87 to 0.92) [29]. The TSK is a questionnaire with 17-items to assess kinesiophobia, with a total score ranging from 17 to 68 points [30]. This tool has suitable reliability (intraclass correlation coefficient 0.93) [26] and a suitable correlation with depressive and catastrophic symptoms [30]. Subjects with TSK scores >37 points are considered subjects with high fear [30].

Participants were assessed in a pain-free period for movement analysis by a trained examiner blinded to the individual’s condition. The Trigno sensors were firmly fixed with adhesive tape bilaterally after proper skin cleaning (cleaned with alcohol/trichotomized when necessary). Electrodes were placed according to the standard instructions at the sternocleidomastoid [31], anterior scalene [31], splenius capitis [32], and upper trapezius [33] muscles. Participants were seated at MCU and fixed firmly with belts. They were asked to perform three repetitions for each cervical movement: flexion, extension, left/right lateral flexions, and left/right rotations in a random sequence. They were instructed to complete the total movement in about 4 s, following audio feedback, in order to obtain similar intervals and velocities to analyze EMG amplitude data. There was a 15 s interval between the repetitions and a one-minute interval between each neck movement. The presence of pain in the neck or the head was assessed using the NPRS immediately after each measurement.

### 2.4. Data Processing

Data were analyzed using a custom MATLAB code (MathWorks, Natick, MA, USA). Kinematic data were filtered with a 4th order low-pass filter with a cutoff frequency of 10 Hz. The peak angle determined maximal cervical ROM, and the angular velocity was calculated based on the mean angular velocity from the beginning to peak angle. ROM and mean angular velocity were reported for each movement separately to be consistent with the EMG data report. However, we do not have biological plausibility to assume a specific laterality restriction in patients with migraines. Moreover, reduced ROM has been inconsistently reported for patients with migraines in all planes [9,10]. So, to provide a reasonable variable for clinical application, we also reported: the sum of the six cervical ROM, which will be named as total cervical ROM, and the average between the six angular velocities named as the mean angular velocity of cervical movement.

Despite the time constraint to perform the active movements, there were still differences among groups at the mean angular velocity. Consequently, we were not able to calculate the signal amplitude of the EMG [19,34]. EMG data were filtered with a 4th order with band-pass filtering 10–950 Hz [35]. Onset muscles were determined when the EMG signal exceeded a 2SD threshold from more than a 25 ms window. These thresholds were determined visually after pilot trials based on recommendations of Hodges et al. [35]. A few trials in which we observed signal interference due to electrode movement were excluded. Detection of the onset and offset of muscle activities during each movement was then quantified in terms of percentage of activation duration to represent temporal characteristics of the muscle activity [20]. A 100% activation indicates that the muscle was active all the time [20].

### 2.5. Statistical Analysis

Normality of data was verified using the Shapiro–Wilk test and observation of residuals distribution on histograms. Means, standard deviations, and frequencies were calculated to describe the variables. Clinical and demographic data, cervical ROM, and angular velocity were compared among the three groups using a one-way analysis of variance (ANOVA) with a Bonferroni post hoc test. The frequency of self-reported neck pain was compared among groups using the chi-square test. Pearson’s correlation was used to verify the association of mean angular velocity of cervical movement and total cervical ROM with headache features (years with migraine, frequency, and intensity), NDI, and TSK scores. Correlation values less than 0.40 indicated a weak correlation, 0.40 to 0.69, moderate, and more than 0.70, strong correlation [36].

Comparison among control, episodic migraine, and chronic migraine groups were performed first. In the case of any significant difference, analysis of subgroups considered by the history of neck pain and the presence of pain during the active movements were also carried out as covariates. For that, a two-way ANOVA with a Bonferroni post hoc test was used.

Multivariate ANOVA was used to compare the percentage of activation duration of all cervical muscles during each movement among groups.

SPSS software (version 20.0, SPSS Inc, Chicago, IL, USA) was used for all statistical analyses adopting a significance level of 0.05.

## 3. Results

### 3.1. Demographics

Of 103 potential eligible individuals, 6 were excluded due to comorbid headache diagnosis, 4 had a history of neck trauma, 2 had received recent anesthetic blocks, and 10 were unavailable to attend the evaluation. Accordingly, the final sample consisted of 27 women with episodic migraine, 27 women with chronic migraine, and 27 headache-free women as controls.

All groups presented similar age (F_(2,80)_ = 1.39 *p* = 0.26) and body mass index (F_(2,80)_ = 0.94, *p* = 0.40). Both episodic and chronic migraine groups exhibited greater prevalence of self-reported neck pain (X^2^ = 16.40, *p* < 0.001) than control group. Significant differences among groups were observed for frequency of neck pain (F_(2,45)_ = 3.42, *p* = 0.04), NDI (F_(2,44)_ = 9.22, *p* < 0.001), and TSK (F_(2,80)_ = 8.48, *p <* 0.001) scores. The chronic migraine group exhibited higher frequency of neck pain than the episodic migraine group (*p* = 0.04), and higher related disability than the control group (*p* < 0.001). The TSK scores were higher in both episodic (*p* = 0.001) and chronic migraine groups (*p* = 0.004) when compared with the control group (Table 1). High fear was identified in 15% of the control group (*n* = 4), 48% of the episodic migraine group (*n* = 13) and 52% of the chronic migraine group (*n* = 14).

### 3.2. Cervical ROM and Angular Velocity

Total cervical ROM differed among groups (F_(2,80)_ = 5.61, *p <* 0.01). Lower ROM could be observed for both episodic (mean difference: 30.52°, *p* = 0.01) and chronic migraine (mean difference: 30.07°, *p* = 0.02) groups compared to controls. When the movements were analyzed separately, differences were observed for left lateral flexion (F_(2,80)_ = 5.05, *p* = 0.009), and right rotation. (F_(2,80)_ = 4.24, *p* = 0.02): chronic migraine women showed less left lateral flexion than controls (*p* = 0.007), whereas episodic migraine women showed less right rotation (*p* = 0.03, Table 2). Differences in right lateral flexion (F_(2,80)_ = 3.26, *p* = 0.04), and left rotation (F_(2,80)_ = 3.14. *p* = 0.04) were observed, but with no significant pairwise comparisons after the adjustments for multiple comparisons were seen (*p* > 0.05, Table 2). No differences for flexion (F_(2,80)_ = 1.38, *p* = 0.25), and extension (F_(2,80)_ = 3.11, *p* = 0.05, Table 2) among the groups were observed.

Subgroup analysis considering the history of neck pain revealed a main effect of group for the total range of motion (F_(2,80)_ = 4.19, *p* = 0.02), but no significant differences for the history of neck pain or the interaction between group and neck pain (*p* > 0.05, Table S1). When groups were stratified by the presence of neck pain during active movement, a group * neck pain interaction was verified for neck flexion (F_(2,__75__)_ = 3.18, *p* = 0.047, Table S2): women with chronic migraine and neck pain during cervical motion present lower cervical ROM than those with chronic migraine but without pain during cervical ROM (*p* = 0.02). The chronic migraine group with neck pain during cervical ROM exhibited lower cervical ROM than the control group with neck pain during cervical flexion (*p* = 0.04).

Mean angular velocity of cervical movement was different among groups (F_(2,80)_ = 6.28, *p* = 0.003). Both groups with migraine presented lower angular velocity than controls, with a mean difference of 3.93°/s for episodic migraine (*p* = 0.02) and 4.51°/s for chronic migraine (*p* < 0.01). When the movements were analyzed separately, angular velocity was different in flexion (F_(2,80)_ = 4.06, *p* = 0.02), right lateral flexion (F_(2,80)_ = 4.66, *p* = 0.01), left lateral flexion (F_(2,80)_ = 4.75, *p* = 0.01), and left rotation (F_(2,80)_ = 6.19, *p* = 0.003). Angular velocity was reduced in chronic migraine as compared to controls for flexion (*p* = 0.04), right (*p* = 0.01) and left (*p* = 0.01) lateral flexion (Table 2). Episodic (*p* = 0.01) and chronic (*p* = 0.006) migraine groups showed lower angular velocity than controls for left rotation (Table 2). No differences were found for extension (F_(2,__75__)_ = 2.75, *p* = 0.07) and right rotation (F_(2,80)_ = 2.66, *p* = 0.07).

The subgroups analysis of history of neck pain showed a main effect of group for right lateral flexion (F_(2,80)_ = 4.78, *p* = 0.01) and right rotation (F_(2,80)_ = 5.19, *p* = 0.008) but no difference related to the history of neck pain neither the interaction of them (*p* > 0.05, Table S3). Similar findings were obtained from the subgroup analysis considering neck pain experienced during cervical ROM. We only found the main effects of the group for flexion (F_(2,80)_ = 4.71, *p* = 0.01, Table S4) without any significant differences for neck pain during the test or the interaction between these two factors (*p* > 0.05).

### 3.3. Percentage of Activation

**Figure 2:**
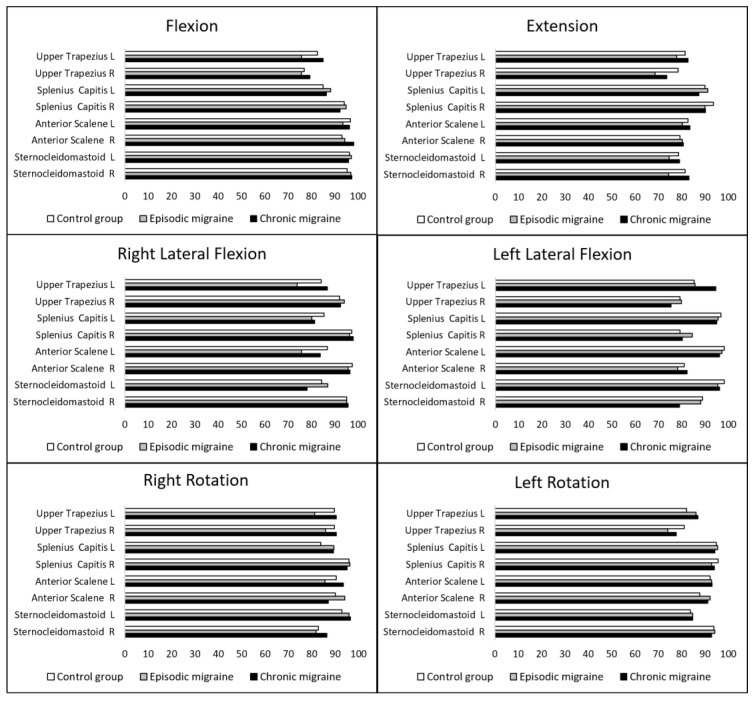
Mean percentage of activation of cervical muscles during active neck movements. R: **right**; L: **left**.

Figure 2 displays the mean percentage of activation of all cervical muscles assessed during active cervical ROM. No differences among groups for any muscle, regardless of being agonist/antagonist, during flexion (F_(16,34)_ = 0.744; *p* = 0.73), extension (F_(16,58)_ = 0.949; *p* = 0.52), right lateral flexion (F_(16,46)_ = 1.324; *p* = 0.22), left lateral flexion (F_(16,42)_ = 1.089; *p* = 0.39), right rotation (F_(16,34)_ = 1.314; *p* = 0.25), or left rotation (F_(16,56)_ = 0.472; *p* = 0.95).

### 3.4. Correlations

Table 3 presents the correlation analysis. Weak negative correlations were observed between total cervical ROM and NDI and TSK scores (*p* < 0.05). Weak-to-moderate negative correlations were observed between mean angular velocity of cervical movement and NDI and TSK scores (*p* < 0.05). No significant correlation was observed between headache features and total cervical ROM or mean angular velocity of cervical movements (*p* < 0.05).

## 4. Discussion

The current study revealed that women with migraines present reduced total cervical ROM and angular velocity during neck active movements compared to controls. In general, those variables were not influenced by the history of neck pain or pain evoked with movement. Differences in the angular velocity were more frequently observed in those with chronic migraines. Cervical ROM and angular velocity were weakly correlated with neck-related disability and kinesiophobia in women with migraines but not with headache features. Finally, the duration of neck muscle activation did not differ between groups for any active movements, regardless of whether agonist or antagonist. These results partially confirmed the study hypotheses.

Our total cervical ROM results agree with a previous study [15], showing reduced total mobility in migraine patients compared to controls, but no differences between episodic and chronic migraineurs. The results of the separate movements may be misinterpreted as a preferred directional restriction. However, currently, there is no plausibility to assume any lateral preference. Moreover, data from two recent systematic reviews with meta-analyses reinforce that any specific side or plane restriction might be random. Liang et al. revealed a reduction in cervical ROM in the sagittal and frontal planes [9], whereas Szikszay et al. observed lower ROM in sagittal and transverse planes, comparing migraineurs and controls [10].

This is the first study analyzing neck angular velocity in migraineurs, revealing a significant reduction in patients with migraine, especially those with the chronic form. However, we should recognize that the mean angular velocity observed in this study does not represent a self-paced velocity that subjects use during their daily activity. The time constraint of our experiment may have forced the participants to adopt a slow movement. Nevertheless, even under the same experimental circumstances, the migraine groups performed the active neck movements more slowly than the controls. It agrees with the lower velocity (peak or average) observed for patients with neck pain when performing self-paced active neck movements [18,37]. Vikne et al. [19] assessed patients with chronic whiplash-associated disorders adopting slow, preferred, and maximal speed to move their head and neck. Lower average velocity was also observed for the patients compared to controls to perform cervical extension and flexion back to the neutral position regardless of the speed assessed [19]. For the cervical flexion, differences were observed only at the maximal speed [19]. Future studies may expand the knowledge about kinematic variables of active neck movements in migraineurs using preferred speed, as maximal speed would not be appropriate for these patients considering the high frequency of vestibular symptoms [38].

Several hypotheses can be raised to speculate the mechanisms behind the lower angular velocity observed in the migraine groups, including the possibility of a combination of them. Subjects with migraines avoid moving their head during a migraine attack [17], so if they perpetuate their behavior even during interictal phases (out of migraine attack), it could facilitate kinematic alterations. Indeed, half of our migraine groups were classified as high fear subjects according to TKS scores, which is the proportion expected among migraineurs [16]. In individuals with neck pain, kinesiophobia presented negative weak-to-moderate associations with kinematic variables [39]. However, the correlations observed in our sample between TKS scores and angular velocity were too low to be clinically highlighted and justify our findings.

Another aspect that could contribute would be the higher frequency of migraine, as the chronic migraine group seems to be more affected when we consider the movements separately. However, the absence of a significant correlation between the frequency of headache and the mean angular velocity suggests that this relationship may not have a significant role in our findings.

In addition to the behavioral and headache frequency hypotheses, there is also the potential contribution of a cervical mechanoreceptor dysfunction to the impairment of kinematic performance [18]. In this context, Meise et al. observed that patients with chronic migraine presented altered cervical proprioception, and the presence of neck pain did not modify it [40]. As no data regarding cervical proprioception were collected, we cannot confirm nor discard this hypothesis. It might be a subject to be explored in further studies.

The combination of lesser active ROM with low velocity might point toward a change in motor control strategies by increasing coactivation of neck muscles to avoid pain or maintain cervical stability [41]. In contrast to our findings, altered superficial muscle activity has been previously reported in patients with migraines [21,23] through a higher activity of extensor muscles during the flexion when compared to healthy controls. However, in those studies, electromyographic activity was assessed during isometric contractions or low-load craniocervical flexion movement. In addition, in the current study, we could not analyze muscle coactivation due to the between-group differences in angular velocity, making a direct comparison between studies difficult. Therefore, our data suggest that the performance of active neck movements is not associated with an altered muscle activation time. It may be justified by the low effort demanded during the task or by its similarity to cervical movements performed frequently during daily routine. Similarly, individuals with migraines did not also differ from controls on muscle activity during the endurance test with submaximal contractions [42]. Current and previous data suggest a complex adaptation of motor control patterns of the cervical musculature in patients with migraines.

The analysis of movement and muscle pattern recruitment during active cervical movements has been analyzed in individuals with chronic neck pain. Previous studies have revealed a reduced range of motion, lower velocity, and increased co-contraction ratio in cervical muscles [18,20,41]. Despite a higher prevalence of neck pain in migraine groups in contrast to the control group, our results were not altered by the history of neck pain or neck pain induced during movement. These findings support the hypothesis that one of the migraine characteristics might be an alteration in the efferent system that affects motor control and mobility of the craniocervical area since some symptoms and signs of cervical dysfunction are related to the migraine itself and not dependent on the presence of neck pain [43].

Nevertheless, the role of neck pain in cervical mobility cannot be totally excluded, whereas we observed in those individuals with self-reported neck pain weak correlations between neck-related disability with cervical ROM and angular velocity. Similar results were previously observed in individuals with migraines [14] and neck pain [39]. For clinicians, we reinforce the importance of cervical assessment in patients with migraines, regardless of the presence of neck pain and the assessment of psychosocial aspects, since they could be negative factors in treatment success. From a scientific perspective, cervical motor control in patients with migraines should be investigated during functional tasks to understand the impact of symptoms or signs of musculoskeletal dysfunction in daily activities. The role of kinesiophobia on musculoskeletal deficits associated with migraine also needs to be better explored since it could be an anticipatory behavior of fear-avoidance to potential pain. Future studies may also investigate a potential relationship between hypervigilance and angular velocity in patients with migraines.

Finally, we recognize some limitations of the current study. Since our sample consisted of only women, the results should not be generalized to men with migraines. In addition, due to the study design, we cannot infer any causal relationship between the factors. Head and neck postures were not assessed, and the altered spine alignment could influence the motor strategies to perform the active cervical movement. Finally, although the TSK had revealed differences in kinesiophobia between patients with migraine and controls, it is not a validated tool in the migraine population. Despite these limitations, this is the first study investigating muscle activity during active neck movements and neck kinematic aspects in patients with migraines, adding that the velocity of the active cervical movement may be as affected as its range of motion. Moreover, it highlights that the history of neck pain or the presence of neck pain during the task seems to exhibit little or no influence on these reduced parameters. However, psychosocial aspects may also contribute to them.

## 5. Conclusions

Women with episodic and chronic migraines presented lower total cervical ROM and mean angular velocity during neck active movements when compared with headache-free controls. Total cervical ROM and mean angular velocity were negative and weakly correlated to neck disability and kinesiophobia, but not to headache features. No differences were observed for the percentage of activation of both neck flexors and extensors acting as antagonists or as agonists during active neck movements.

## Figures and Tables

**Table 1:** Mean, standard deviation, and frequency of sample sociodemographic characteristics and clinical features.

	Control Group (*n* = 27)	Episodic Migraine (*n* = 27)	Chronic Migraine (*n* = 27)
Age (years)	31.2 (9.17)	33.0 (9.05)	35.5 (10.27)
BMI (kg/cm²)	25.0 (4.00)	23.7 (3.89)	23.9 (2.95)
Years with migraine	-	14.1 (8.33)	18.1 (11.55)
Migraine frequency (days/month)	-	6.7 (3.29)	24.5 (5.66)
Migraine intensity (NPRS)	-	7.6 (1.49)	8.0 (1.57)
Self-report of neck pain †	7 (25.9%)	18 (66.7%)	21 (77.8%)
Years with neck pain	3.6 (2.17)	9.2 (4.58)	8.4 (6.82)
Neck pain frequency (days/month)	13.9 (11.34)	12.5 (10.70)	20.5 (8.64) **
Neck pain intensity (NPRS)	4.4 (1.27)	5.5 (2.01)	5.8 (2.18)
NDI score	11.1 (11.65)	24.9 (11.58)	35.1 (14.66) *
TSK score	28.7 (7.53)	36.1 (8.05) *	37.2 (9.14) *

* *p* < 0.05 vs. control group; ** *p* < 0.05 vs. episodic migraine group; † chi-square test *p* < 0.05; BMI: body mass index; NPRS: numeric pain rating scale; NDI: Neck Disability Index; TSK: Tampa Scale for Kinesiophobia.

**Table 2:** Mean and standard deviation of cervical range of motion angle and angular velocity.

	Control Group (*n* = 27)	Episodic Migraine (*n* = 27)	Chronic Migraine (*n* = 27)
Cervical range of motion (degrees)			
Total range of motion	310.7 (28.39)	280.21 (37.01) *	280.65 (47.39) *
Flexion	58.0 (6.91)	53.8 (7.68)	55.5 (12.68)
Extension	59.7 (7.49)	53.9 (9.71)	55.2 (9.59)
Right lateral flexion	51.6 (8.26)	46.3 (7.42)	46.6 (9.80)
Left lateral flexion	51.0 (7.68)	46.1 (8.26)	43.4 (10.53) *
Right rotation	73.0 (8.50)	65.1 (9.29) *	65.9 (14.40)
Left rotation	68.9 (11.93)	61.3 (12.21)	60.7 (15.81)
Angular velocity (degrees/s)			
Mean angular velocity of cervical moviment	26.60 (4.62)	22.67 (4.72) *	22.09 (5.84) *
Flexion	27.17 (5.43)	23.2 (5.07)	23.0 (7.34) *
Extension	25.33 (5.51)	22.7 (5.78)	22.2 (4.57)
Right lateral flexion	23.07 (5.31)	19.7 (4.90)	19.2 (5.80) *
Left lateral flexion	22.9 (5.77)	19.2 (5.10)	18.8 (6.13) *
Right rotation	30.4 (5.82)	26.2 (6.48)	26.4 (9.44)
Left rotation	30.7 (5.86)	25.0 (6.57) *	25.3 (8.04) *

* *p* < 0.05 vs. control group.

**Table 3:** Pearson’s correlations (r) and 95% confidence intervals of angular velocity and cervical range of motion angle with both Neck Disability Index (NDI) and Tampa Scale for Kinesiophobia (TSK) scores and with headache features.

	Total Cervical Range of Motion (°)	Angular Velocity (°/s)
Total sample (*n* = 81)		
NDI scores	−0.25 * (−0.48 to −0.04)	−0.28 (−0.50 to −0.06) *
TSK scores	−0.30 * (−0.51 to −0.08)	−0.40 (−0.60 to −0.19) **
Participants with migraine (*n* = 54)		
Years with migraine	−0.003 (−0.32 to 0.32)	0.01 (−0.28 to 0.31)
Migraine frequency (days/month)	0.14 (−0.16 to 0.47)	0.04 (−0.25 to 0.34)
Migraine intensity (NPRS)	0.13 (−0.39 to 1.07)	0.02 (−0.64 to 0.74)

* *p* < 0.05; ** *p* < 0.01.

## Data Availability

The data presented in this study are available on request from the corresponding author. The data are not publicly available due to containing information that could compromise the privacy of research participants.

## Supplementary Materials

The following are available online at https://www.mdpi.com/article/10.3390/jcm10173805/s1, Table S1: Cervical range of motion (degrees) of the three groups stratified according to the history of neck pain (mean and standard deviation), Table S2: Cervical range of motion (degrees) of the three groups stratified according to the presence of neck pain during active movement (mean and standard deviation, Table S3: Angular velocity (degrees/s) of the three groups stratified according to the history of neck pain (mean and standard deviation), Table S4: Angular velocity (°/s) of the three groups stratified according to the presence of neck pain during active movement (mean and standard deviation).

## References

[1] Steiner T.J., Stovner L.J., Jensen R., Uluduz D., Katsarava Z. (2020). Lifting The Burden: The Global Campaign against Headache. Migraine remains second among the world’s causes of disability, and first among young women: Findings from GBD2019. J. Headache Pain.

[2] Headache Classification Committee of the International Headache Society (IHS) (2018). The International Classification of Headache Disorders, 3rd ed. Cephalalgia.

[3] Burch R.C., Buse D.C., Lipton R.B. (2019). Migraine: Epidemiology, Burden, and Comorbidity. Neurol. Clin..

[4] Lipton R.B., Fanning K.M., Buse D.C., Martin V.T., Reed M.L., Adams A.M., Goadsby P.J. (2018). Identifying Natural Subgroups of Migraine Based on Comorbidity and Concomitant Condition Profiles: Results of the Chronic Migraine Epidemiology and Outcomes (CaMEO) Study. Headache.

[5] Goadsby P.J., Holland P.R., Martins-Oliveira M., Hoffmann J., Schankin C., Akerman S. (2017). Pathophysiology of Migraine: A Disorder of Sensory Processing. Physiol. Rev..

[6] Aguila M.R., Rebbeck T., Pope A., Ng K., Leaver A.M. (2018). Six-month clinical course and factors associated with non-improvement in migraine and non-migraine headaches. Cephalalgia.

[7] Ford S., Calhoun A., Kahn K., Mann J., Finkel A. (2008). Predictors of disability in migraineurs referred to a tertiary clinic: Neck pain, headache characteristics, and coping behaviors. Headache.

[8] Charles A. (2018). The pathophysiology of migraine: Implications for clinical management. Lancet Neurol..

[9] Liang Z., Galea O., Thomas L., Jull G., Treleaven J. (2019). Cervical musculoskeletal impairments in migraine and tension type headache: A systematic review and meta-analysis. Musculoskelet. Sci. Pract..

[10] Szikszay T.M., Hoenick S., von Korn K., Meise R., Schwarz A., Starke W., Luedtke K. (2019). Which Examination Tests Detect Differences in Cervical Musculoskeletal Impairments in People With Migraine? A Systematic Review and Meta-Analysis. Phys. Ther..

[11] Oliveira-Souza A.I.S., Florencio L.L., Carvalho G.F., Fernández-de-Las-Peñas C., Dach F., Bevilaqua-Grossi D. (2019). Reduced flexion rotation test in women with chronic and episodic migraine. Braz. J. Phys. Ther..

[12] Bevilaqua-Grossi D., Pegoretti K.S., Goncalves M.C., Speciali J.G., Bordini C.A., Bigal M.E. (2009). Cervical mobility in women with migraine. Headache.

[13] Ferracini G.N., Florencio L.L., Dach F., Bevilaqua-Grossi D., Palacios-Ceña M., Ordás-Bandera C., Chaves T.C., Speciali J.G., Fernández-de-las-Peñas C. (2017). Musculoskeletal disorders of the upper cervical spine in women with episodic or chronic migraine. Eur. J. Phys. Rehabil. Med..

[14] Carvalho G.F., Chaves T.C., Gonçalves M.C., Florencio L.L., Braz C.A., Dach F., Fernández-de-las-Peñas C., Bevilaqua-Grossi D. (2014). Comparison between neck pain disability and cervical range of motion in patients with episodic and chronic migraine: A cross-sectional study. J. Manip. Physiol. Ther..

[15] Luedtke K., Starke W., May A. (2018). Musculoskeletal dysfunction in migraine patients. Cephalalgia.

[16] Benatto M.T., Bevilaqua-Grossi D., Carvalho G.F., Bragatto M.M., Pinheiro C.F., Lodovichi S.S., Dach F., Fernández-de-las-Peñas C., Florencio L.L. (2019). Kinesiophobia Is Associated with Migraine. Pain Med..

[17] Martins I.P., Gouveia R.G., Parreira E. (2006). Kinesiophobia in migraine. J. Pain.

[18] Salehi R., Rasouli O., Saadat M., Mehravar M., Negahban H., Shaterzadeh M.J. (2021). Cervical movement kinematic analysis in patients with chronic neck pain: A comparative study with healthy subjects. Musculoskelet. Sci. Pract..

[19] Vikne H., Bakke E.S., Liestøl K., Engen S.R., Vøllestad N. (2013). Muscle activity and head kinematics in unconstrained movements in subjects with chronic neck pain; cervical motor dysfunction or low exertion motor output?. BMC Musculoskelet. Disord..

[20] Tsang S.M., Szeto G.P., Lee R.Y. (2014). Altered spinal kinematics and muscle recruitment pattern of the cervical and thoracic spine in people with chronic neck pain during functional task. J. Electromyogr. Kinesiol..

[21] Benatto M.T., Florencio L.L., Bragatto M.M., Lodovichi S.S., Dach F., Bevilaqua-Grossi D. (2019). Extensor/flexor ratio of neck muscle strength and electromyographic activity of individuals with migraine: A cross-sectional study. Eur. Spine J..

[22] Florencio L.L., Oliveira A.S., Lemos T.W., Carvalho G.F., Dach F., Bigal M.E., Falla D., Fernández-de-las-Peñas C., Bevilaqua-Grossi D. (2016). Patients with chronic, but not episodic, migraine display altered activity of their neck extensor muscles. J. Electromyogr. Kinesiol..

[23] Florencio L.L., Oliveira A.S., Carvalho G.F., Tolentino G.A., Dach F., Bigal M.E., Fernández-de-las-Peñas C., Bevilaqua-Grossi D. (2015). Cervical Muscle Strength and Muscle Coactivation During Isometric Contractions in Patients With Migraine: A Cross-Sectional Study. Headache.

[24] Chiu T.T., Sing K.L. (2002). Evaluation of cervical range of motion and isometric neck muscle strength: Reliability and validity. Clin. Rehabil..

[25] Cook C., Richardson J.K., Braga L., Menezes A., Soler X., Kume P., Zaninelli M., Socolows F., Pietrobon R. (2006). Cross-cultural adaptation and validation of the Brazilian Portuguese version of the Neck Disability Index and Neck Pain and Disability Scale. Spine (Phila Pa 1976).

[26] Souza F.S., Marinho C.S., Siqueira F.B., Maher C.G., Costa L.O. (2008). Psychometric testing confirms that the Brazilian-Portuguese adaptations, the original versions of the Fear-Avoidance Beliefs Questionnaire, and the Tampa Scale of Kinesiophobia have similar measurement properties. Spine (Phila Pa 1976).

[27] Vernon H. (2008). The Neck Disability Index: State-of-the-art, 1991-2008. J. Manip. Physiol. Ther..

[28] Jorritsma W., Dijkstra P.U., de Vries G.E., Geertzen J.H., Reneman M.F. (2012). Detecting relevant changes and responsiveness of Neck Pain and Disability Scale and Neck Disability Index. Eur. Spine J..

[29] Schellingerhout J.M., Verhagen A.P., Heymans M.W., Koes B.W., de Vet H.C., Terwee C.B. (2012). Measurement properties of disease-specific questionnaires in patients with neck pain: A systematic review. Qual. Life Res..

[30] Vlaeyen J.W., Kole-Snijders A.M., Rotteveel A.M., Ruesink R., Heuts P.H. (1995). The role of fear of movement/(re)injury in pain disability. J. Occup. Rehabil..

[31] Falla D., Dall’Alba P., Rainoldi A., Merletti R., Jull G. (2002). Location of innervation zones of sternocleidomastoid and scalene muscles--a basis for clinical and research electromyography applications. Clin. Neurophysiol..

[32] Joines S.M., Sommerich C.M., Mirka G.A., Wilson J.R., Moon S.D. (2006). Low-level exertions of the neck musculature: A study of research methods. J. Electromyogr. Kinesiol..

[33] Surface ElectroMyoGraphy for the Non-Invasive Assessment of Muscles. http://www.seniam.org.

[34] Tsang S.M., Szeto G.P., Lee R.Y. (2016). Relationship between neck acceleration and muscle activation in people with chronic neck pain: Implications for functional disability. Clin. Biomech..

[35] Hodges P.W., Bui B.H. (1996). A comparison of computer-based methods for the determination of onset of muscle contraction using electromyography. Electroencephalogr. Clin. Neurophysiol..

[36] Domholdt E. (2000). Physical Therapy Research: Principles and Applications.

[37] Sarig Bahat H., Chen X., Reznik D., Kodesh E., Treleaven J. (2015). Interactive cervical motion kinematics: Sensitivity, specificity and clinically significant values for identifying kinematic impairments in patients with chronic neck pain. Man. Ther..

[38] Carvalho G.F., Vianna-Bell F.H., Florencio L.L., Pinheiro C.F., Dach F., Bigal M.E., Bevilaqua-Grossi D. (2019). Presence of vestibular symptoms and related disability in migraine with and without aura and chronic migraine. Cephalalgia.

[39] Sarig Bahat H., Weiss P.L., Sprecher E., Krasovsky A., Laufer Y. (2014). Do neck kinematics correlate with pain intensity, neck disability or with fear of motion?. Man. Ther..

[40] Meise R., Lüdtke K., Probst A., Stude P., Schöttker-Königer T. (2019). Zervikaler “joint position error” bei Kopfschmerzen: Systematische Literaturübersicht und empirische Daten bei chronischer Migräne [Joint position error in patients with headache: Systematic review of the literature and experimental data for patients with chronic migraine]. Schmerz.

[41] Sjölander P., Michaelson P., Jaric S., Djupsjöbacka M. (2008). Sensorimotor disturbances in chronic neck pain--range of motion, peak velocity, smoothness of movement, and repositioning acuity. Man. Ther..

[42] Florencio L.L., Oliveira A.S., Will-Lemos T., Pinheiro C.F., Marçal J.C.D.S., Dach F., Fernández-de-Las-Peñas C., Bevilaqua-Grossi D. (2021). Muscle endurance and cervical electromyographic activity during submaximal efforts in women with and without migraine. Clin. Biomech..

[43] Bragatto M.M., Bevilaqua-Grossi D., Benatto M.T., Lodovichi S.S., Pinheiro C.F., Carvalho G.F., Dach F., Fernández-de-las-Peñas C., Florencio L.L. (2019). Is the presence of neck pain associated with more severe clinical presentation in patients with migraine? A cross-sectional study. Cephalalgia.

