# Supplementary material for: Neck Active Movements Assessment in Women with Episodic and Chronic Migraine
Source: J Clin Med. 2021 Aug 25;10(17):3805. doi: 10.3390/jcm10173805 (PMC8432227; doi:10.3390/jcm10173805)
Supplement: Supplementary file 1 [file jcm-10-03805-s001.zip › jcm-1263355-supplementary.pdf]

Table S1. Cervical range of motion (degrees) of the three groups stratified according to the history of neck pain (mean and standard deviation)

	Control group (27)		Episodic migraine (27)		Chronic migraine (27)	
	**No neck pain (20)**	**Neck pain (7)**	**No neck pain (9)**	**Neck pain (18)**	**No neck pain (6)**	**Neck pain (21)**
Total range of motion*†	309.28 (27.90)	314.86 (31.62)	288.63 (43.20)	276.00 (34.05)	282.50 (32.76)	280.12 (51.47)
Flexion	56.90 (7.09)	61.28 (5.55)	57.00 (9.88)	52.20 (6.00)	59.94 (11.12)	54.19 (13.04)
Extension	59.51 (7.24)	60.42 (8.71)	54.29 (9.25)	53.77 (10.19)	53.11 (7.53)	55.80 (10.18)
Right lateral flexion	51.85 (8.58)	50.90 (7.87)	47.70 (9.30)	45.54 (6.45)	47.16 (9.98)	46.46 (9.98)
Left lateral flexion	51.36 (8.48)	49.90 (5.11)	47.81 (8.60)	45.18 (8.19)	43.72 (3.22)	43.30 (11.89)
Right rotation	73.15 (8.51)	72.76 (9.12)	66.62 (8.70)	64.33 (9.71)	66.77 (20.08)	65.65 (12.98)
Left rotation	68.35 (11.46)	70.47 (14.02)	62.88 (14.53)	60.50 (11.24)	58.94 (11.73)	61.17 (17.01)

*p<0.05 chronic migraine vs. control group; † p<0.05 episodic migraine vs. control group

.

Table S2. Cervical range of motion (degrees) of the three groups stratified according to the presence of neck pain during active movement (mean and standard deviation)

	**Control group (27)**		**Episodic migraine (27)**		**Chronic migraine (27)**	
	**No neck pain**	**Neck pain [a]**	**No neck pain**	**Neck pain [b]**	**No neck pain**	**Neck pain [c]**
Total range of motion	NA	NA	NA	NA	NA	NA
Flexion	57.13 (6.50)	65.22 (6.88)	53.91 (7.48)	53.16 (9.95)	58.62 (9.37)	50.10 (16.03) *†
Extension	59.86 (7.71)	58.88 (6.61)	54.83 (10.06)	52.18 (9.28)	54.78 (6.20)	55.66 (12.54)
Right lateral flexion	51.88 (8.28)	48.16 (10.13)	45.97 (7.68)	47.09 (7.07)	45.45 (7.69)	47.87 (11.85)
Left lateral flexion	51.76 (7.63)	44.77 (5.71)	46.09 (7.81)	45.93 (11.08)	41.94 (6.97)	44.55 (12.81)
Right rotation	73.26 (8.67)	70.33 (7.54)	65.88 (9.32)	62.33 (9.44)	67.48 (15.00)	63.91 (14.00)
Left rotation	69.80 (11.93)	57.66 (3.30)	61.68 (11.91)	59.08 (15.60)	63.78 (12.14)	55.40 (20.27)

*p <0.05 vs no neck pain chronic migraine; †p<0.05 vs neck pain control group

[a] number of subjects with neck pain during each cervical movement in the control group: flexion = 3; extension = 3; right lateral flexion = 2; left lateral flexion = 3; right rotation = 2; left rotation = 2. [b] number of subjects with neck pain during each cervical movement in the episodic migraine group: flexion = 4; extension = 9; right lateral flexion = 7; left lateral flexion = 5; right rotation = 6; left rotation = 4. [c] number of subjects with neck pain during each cervical movement in the chronic migraine group: flexion = 10; extension = 13; right lateral flexion = 13; left lateral flexion = 15; right rotation = 12; left rotation = 10. NA = not available, due to the different number of subjects who reported pain during each movement

Table S3. Angular velocity (degrees/s) of the three groups stratified according to the history of neck pain (mean and standard deviation)

	Control group (27)		Episodic migraine (27)		Chronic migraine (27)	
	**No neck pain (20)**	**Neck pain (7)**	**No neck pain (9)**	**Neck pain (20)**	**No neck pain (7)**	**Neck pain (9)**
Mean angular velocity of cervical movements *	27.11 (4.37)	25.12 (5.36)	21.77 (5.88)	23.11 (4.14)	21.10 (4.86)	22.37 (6.16)
Flexion	27.87 (5.22)	26.81 (6.40)	23.44 (6.06)	23.12 (4.68)	23.88 (6.04)	22.76 (7.78)
Extension	34.94 (6.11)	33.43 (6.51)	29.69 (7.18)	28.86 (6.43)	26.31 (14.41)	27.59 (9.30)
Right lateral flexion *	23.40 (5.54)	22.09 (4.83)	18.08 (5.22)	20.45 (4.68)	15.02 (8.42)	19.46 (6.12)
Left lateral flexion	23.37 (5.75)	21.47 (5.99)	18.09 (4.29)	19.71 (5.49)	18.45 (4.36)	17.95 (7.69)
Right rotation	31.44 (5.34)	27.37 (6.44)	25.47 (6.79)	26.66 (6.48)	27.26 (13.86)	26.21 (8.22)
Left rotation *†	31.35 (5.46)	29.00 (7.01)	23.89 (8.50)	25.47 (5.57)	20.67 (9.02)	25.97 (6.82)

*p<0.05 chronic migraine vs. control group; † p<0.05 episodic migraine vs. control group

Table S4. Angular velocity (degrees/s) of the three groups stratified according to the presence of neck pain during active movement (mean and standard deviation)

	**Control group (27)**		**Episodic migraine (27)**		**Chronic migraine (27)**	
	**No neck pain**	**Neck pain [a]**	**No neck pain**	**Neck pain [b]**	**No neck pain**	**Neck pain [c]**
Mean angular velocity of cervical movements	NA	NA	NA	NA	NA	NA
Flexion *†	26.72 (5.54)	30.71 (2.85)	23.66 (4.76)	20.77 (6.86)	24.48 (5.25)	20.50 (9.77)
Extension	25.98 (5.41)	20.08 (3.26)	22.84 (5.96)	22.43 (5.73)	22.54 (4.39)	21.74 (4.88)
Right lateral flexion	23.15 (5.51)	21.97 (0.67)	19.71 (5.09)	19.52 (4.65)	17.03 (6.38)	20.03 (7.11)
Left lateral flexion	23.21 (5.81)	20.23 (5.55)	19.04 (4.44)	19.75 (8.06)	16.78 (7.42)	19.09 (6.76)
Right rotation	30.17 (5.86)	32.98 (6.27)	26.82 (7.09)	24.29 (3.35)	29.26 (9.97)	22.91 (7.72)
Left rotation	31.00 (6.01)	27.58 (1.18)	25.23 (6.36)	23.31 (8.53)	24.60 (9.64)	23.92 (9.06)

*p<0.05 chronic migraine vs. control group; † p<0.05 episodic migraine vs. control group

[a] number of subjects with neck pain during each cervical movement in the control group: flexion = 3; extension = 3; right lateral flexion = 2; left lateral flexion = 3; right rotation = 2; left rotation = 2. [b] number of subjects with neck pain during each cervical movement in the episodic migraine group: flexion = 4; extension = 9; right lateral flexion = 7; left lateral flexion = 5; right rotation = 6; left rotation = 4. [c] number of subjects with neck pain during each cervical movement in the chronic migraine group: flexion = 10; extension = 13; right lateral flexion = 13; left lateral flexion = 15; right rotation = 12; left rotation = 10. NA = not available, due to the different number of subjects who reported pain during each movement
